# Impact and Modifications of In-Hospital Trauma Care Workflow Due to COVID 19 Pandemic: Lessons Learnt for the Future

**DOI:** 10.30476/BEAT.2021.88507

**Published:** 2021-04

**Authors:** Gaurav Kaushik, Ankita Sharma, Dinesh Bagaria, Subodh Kumar, Sushma Sagar, Amit Gupta

**Affiliations:** 1 Division of Trauma Surgery and Critical Care, Jai Prakash Narayan Apex Trauma Centre, AIIMS, New Delhi, India

**Keywords:** Hospital, Trauma Care, COVID-19, Pandemic

## Abstract

**Objective:**

To describe the restructuring in-hospital systems of care at a Level -1 trauma center in India and to analyze an injury volume and patterns for future preparedness as well as to establish a specific injuries preventive measures during health emergencies like COVID-19.

**Methods:**

Data was extracted from a prospectively managed trauma registry at level-1 trauma center in India. We have compared the data in lockdown period with the same day’s number from the pre-lockdown period. Patients were categorized according to age, gender, injury cause, injury place, injury severity, and injury outcome to compare the statistical analysis between two periods.

**Results:**

Total emergency department (ED) trauma footfall decreased significantly by 73% during lockdown period. The injuries result increased significantly due to blunt forces. There was a significant decrease in the major injury of the patient’s percentage. The road traffic injuries (RTIs) in individuals were less than the reported falls number, which increased significantly during lockdown. The less number of patients significantly presented without receiving primary care. Majority of the patients had been transferred by using private cars, police vehicle, and two wheelers during lockdown; however, patients’ less number were transferred significantly by three wheelers as expected. The comparative analysis between quantitative data points shows significant differences in median Injury Severity Score (ISS) and length of stay during lockdown.

**Conclusion:**

This study highlighted that the preparedness should not focus solely on the response to treat infectious disease during health emergencies but also on ensuring access and provision of reasonable quality of care for non-infectious illnesses especially acute conditions like trauma.

## Introduction

The world has witnessed an unprecedented and highly infective disease pandemic; the Novel Coronavirus disease-2019 (COVID -19) with the aurora of 2020 year. India has been one of the most severely affected countries. Over 10 million (total 10667741) confirmed individuals were reported with 156,111 deaths in India until 19^th^ February 2021 [1]. 

The official response was consisted an international and domestic restriction travel and nationwide lockdown around the world. A pandemic of such magnitude created a unique challenge to the healthcare system worldwide and forced countries to make radical reforms in their health delivery systems. The major actions had been taken include routine outpatient services shutdown, closing of almost all elective surgeries, turning specialty centers into dedicated COVID-19 hospitals and reorganizing workforce arrangement to deal with a potential shortage of personal protective equipment (PPE) as well as to minimize the number of healthcare workers (HCWs) exposed to the virus.

Health emergencies did not follow the expected trend during the lockdown period and trauma is one among them. Little is known about how the COVID- 19 and nationwide lockdown have impacted the trauma care units functioning as well as an injury patterns and volume. Few studies from different countries have described small cohorts and concluded that COVID-19 lockdown impact had an outcome in mixed patterns related to an injury [1, 2].

The present study purpose is describing the restructure at in-hospital care systems at a Level -1 trauma center in India and to analyze an injury volume and patterns for future preparedness as well as to establish a preventive measure for specific injuries during a situation like being in COVID-19 pandemic.

## Materials and Methods

The present study was conducted at All India Institute of Medical Sciences (AIIMS), New Delhi, a premier healthcare institute of the country providing the state of the art patient’s care, teaching and research services to the National Capital Region (NCR) and surrounding states comprising of a population around 320 million. In India, a nationwide lockdown was announced on 24^th^ March, 2020 initially for three-weeks but was extended in four phases till 31^st^ May, 2020. The last phase was not as rigorous and some concessions in intra-state and inter-state travel were granted from 18^th^ to 31^st^ May, 2020. The current data has been extracted from a prospectively managed trauma registry as part of a research project which is funded by the Indian Council of Medical Research (ICMR). 

The trained and dedicated data collectors filled the real-time data round the clock. We have compared lockdown period data (25^th^ March through 31^st^ May, 2020) with data of the same day’s number from the pre-lockdown period (15^th^ January through 24^th^ March, 2020). Patients were grouped according to age, gender, injuries causes, injuries places, an injury severity and injuries outcome for comparative analysis between two periods.

Statistical Analysis 

The statistical analyses were performed by using IBM SPSS Statistics, Version 22.0. (IBM Corp, Armonk, NY). Results were presented as median and interquartile range and percentage quantity wherever applicable. The Chi-Square or Fisher’s exact test was used for categorical variables according to the cell frequencies. Man-Whitney test was performed and data presented as median, Interquartile range (IQR) for continues variables. *P*<0.05 was considered statistically significant. 

## Results

**Fig. 1:**
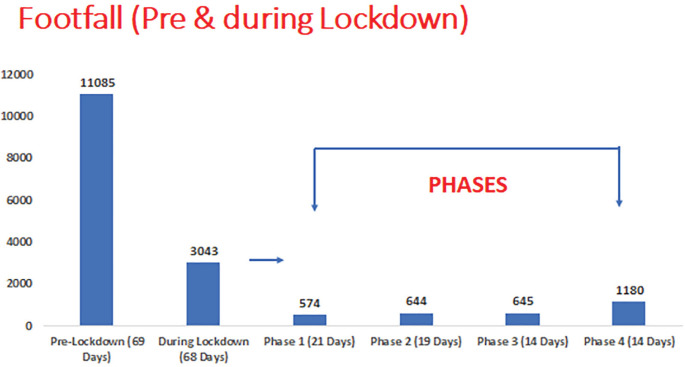
Numbers of visits to trauma emergency services throughout the pre-lockdown and lockdown period

**Fig. 2:**
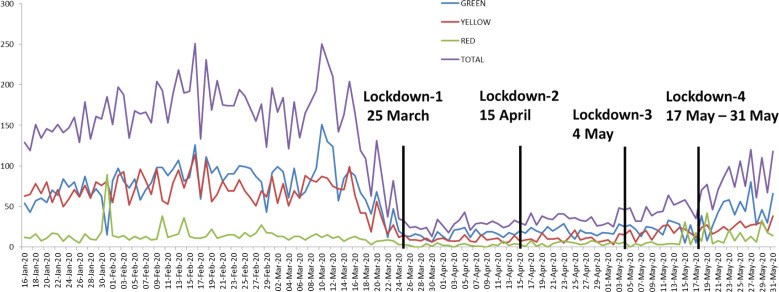
Triage wise comparison of daily emergency department (ED) footfall during pre-lockdown and lockdown periods

**Fig. 3:**
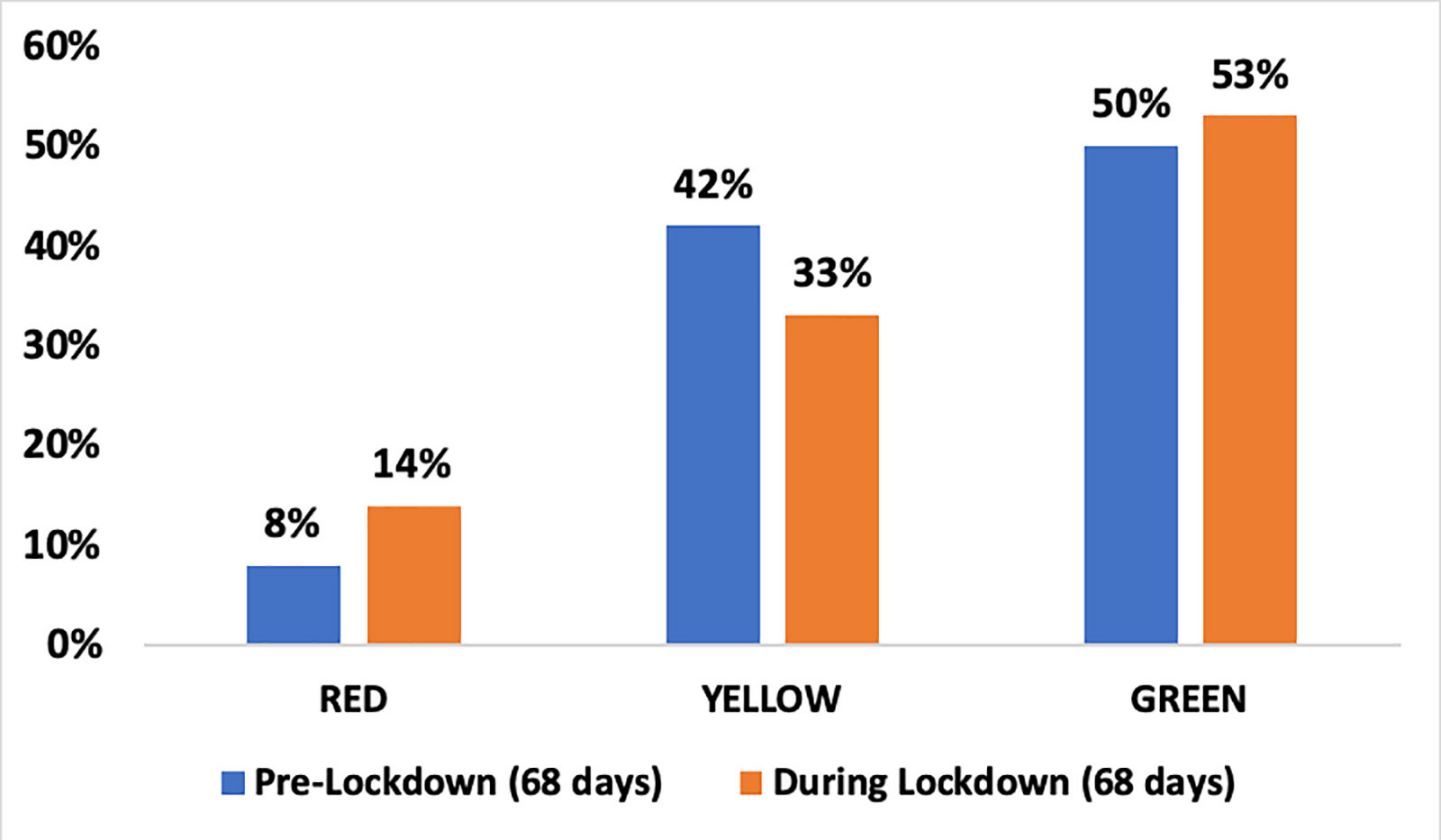
Triage wise breakup of all emergency department (ED) visits during pre-lockdown and lockdown period

Total emergency department (ED) footfall were decreased significantly by 73% (11,085 during pre-lockdown, 3043 during lockdown period) due to trauma (Figures 1 and 2). Among total ED visits, red triage (compromised ABCD) increased and yellow triage (stable ABCD) decreased while green triaged (walking wounded) were remained unchanged (Figure 3). The demographics, injury severity, disposition and outcome were compared between two time periods (Table 1). The age and gender groups’ distribution were remained unchanged. Injuries outcome were increased significantly during lockdown and due to blunt forces. 

**Table 1:** Comparison of demographics, injury severity, mechanism, disposition, and outcome of patients between two time periods

**Parameter**	**Total** **(614)**	**Pre-Lockdown** ** (15 Jan-24 March)** **n=440 (Group 1) n (%)**	**During Lockdown** **25th March–31st May) ** **n=174 (Group 2) n (%)**	***p*** ** value**
Gender				0.552
Male	478	345 (78)	133 (76)	
Female	133	92 (21)	41 (24)	
Transgender	3	03 (1)	0 (0)	
Age Bands (Years)				0.772
0-14	85	63 (14)	22 (13)	
15-64	475	340 (77)	135 (77)	
65+	54	37 (9)	17 (10)	
Dominant type				0.006
Blunt	484	336 (76)	148 (85)	
Penetrating	110	84 (19)	26 (15)	
Mixed	20	20 (5)	0 (0)	
Severity				<0.001
Major ISS^a^>12	130	109 (25)	21 (12)	
Moderate ISS <12	484	331 (75)	153 (88)	
GCS^b^				0.058
3-8	58	37 (8)	21 (12)	
9-12	46	39 (9)	7 (4)	
13-15	510	364 (83)	146 (84)	
Mechanism of injury				
Railway track injury	7	7 (2)	0 (0)	0.200
Assault	42	26 (6)	16 (9)	0.146
Fall	229	151 (34)	78 (45)	0.015
Occupational injury	9	7 (2)	02 (1)	1.000
RTI^c^	293	228 (51)	65 (38)	0.003
Self-harm	5	03 (1)	02 (1)	0.625
Others	29	18 (4)	11 (6)	0.240
Mode of Arrival				
M2W^d^	15	3 (1)	12 (7)	<0.001
M3W^e^	116	112 (25)	4 (2)	<0.001
Private Car	270	170 (39)	100 (57)	<0.001
Ambulance	146	112 (25)	34 (20)	0.121
Police vehicle	61	37 (8)	24 (14)	0.044
Unknown	6	6 (2)	0 (0)	0.191
Primary care received				0.005
Yes	130	106 (24)	24 (14)	
No	484	334 (76)	150 (86)	
Referred				0.008
Yes	129	104 (24)	25 (14)	
No	485	336 (76)	149 (86)	
ED Disposition				0.225
OT^f^	128	84 (19)	44 (25)	
Ward	382	281 (64)	101 (58)	
ICU^g^	104	75 (17)	29 (17)	
Admitting department				
Trauma Surgery	164	115 (26)	49 (28)	0.609
Orthopedics	235	174 (40)	61 (35)	0.303
Neurosurgery	158	119 (27)	39 (22)	0.237
Plastic Surgery	25	19 (4)	6 (4)	0.623
Others (pediatric surgery, emergency medicine etc)	32	13 (3)	19 (11)	<0.001
Hospital Disposition				0.305
Discharge	577	415 (94)	162 (93)	
Death	32	23 (5)	9 (5)	
LAMA^h^	05	02 (1)	3 (2)	

^a^ISS: Injury Severity Score; ^b^GCS: Glasgow Coma Scale; ^c^RTI: Road Traffic Injury; ^d^OT: Operation Theatre; ^e^ICU: Intensive Care Unit; ^f^M2W: Motorized two-wheeler; ^g^M3W: Motorized three-wheeler; ^h^LAMA: Left Against Medical Advice

The road traffic injury (RTI) victims’ number were decreased significantly during lockdown but the proportion of Red Triaged RTI patients was increased. There was also a significant increase in number of ‘falls’ reported during lockdown. Overall, there was a significant decrease in the patients’ percentage who had major trauma (Injury Severity Score, ISS>12) during lockdown. Less patients number were presented significantly without receiving primary care. Most of the patients were transported by using personal cars and motorized two wheelers. Although the transportation of injured victims by ambulances were decreased but police vehicle transportation of such cases were increased. More patients were disposed from ED to operation theatre (OT), though it was not statistically significant. The final outcomes were remained unchanged. Significant differences were found during lockdown in patients admitted to pediatric surgery and emergency medicine as compared to pre-lockdown period. However, no significant difference was observed in admission pattern of trauma surgery, orthopedics and neurosurgery specialties. 

Table 2 shows the comparative analysis between quantitative data points. The Initial vital signs at presentation to ED were remained unchanged. Significant difference in ISS and length of stay was found during lockdown. 

**Table 2:** Comparison of quantitative variables between two time periods (Median, IQR).

	**Pre-Lockdown** **(15 Jan-24 March)** **n=440** **(Group 1)** **Median (IQR** ^a^ **)**	**During Lockdown** **25 March–31 May) ** **n=174** **(Group 2)** **Median (IQR** ^a^ **)**	***p*** ** value**
Age	34 (23)	35 (24)	0.58
Heart Rate	86 (16)	88 (20)	0.80
Resp. Rate	18 (2)	18 (0)	0.96
SpO2	99 (2)	99 (2)	0.98
ISS	9 (7.5)	9 (5)	0.005
Length of Hospital stay (days)	6 (6)	4 (6)	<0.001

^a^IQR: Interquartile Range

COVID-19 Positive Patients

Out of 174 admitted patients (12%), 21 were examined and tested of positive COVID-19 by using RT-PCR after clinical evaluation and during the study period. There were two deaths (one COVID-19 related).

The Functioning Impact of Trauma Surgery Unit

The trauma individuals have been increasing after the initial fall of Trauma ED footfall during the nationwide lockdown and had the COVID-19 positive patient’s graph. The pandemic has created a difficult situation for healthcare workers especially in acute care setting which require rapid assessment and potentially risky procedures, especially emergent surgeries in patients who’s COVID-19 status is unknown. We need more workforce to manage an overwhelming number of sick patients; therefore, more people means more exposure. On the other hand, and at any point of time, we need to have only the bare minimum number of workers required to execute the task. 

The trauma care team was divided into four groups to maintain an effective pool of reserve workers. The ED team who were engaged in the first contact with a trauma victim, named the frontline team. They don the highest-Level PPE at all times and were responsible for the immediate management of the incoming patient and irrespective of their COVID-19 status. The In-patient’s team (ward and intensive care unit) were the next group. After the initial management in the ED, they look after the patients who have been admitted except for immediate life threatening injuries and/ or limb-threatening injuries (if injuries do not treated timely will have an extremity amputations potential such as extremity vascular injuries, mangled extremities, extremity injuries with significant soft tissue loss and near total amputations). All ED patients were screened for Sars-Cov-2 before being handed over to the Ward and ICU team. They don a Level-1 PPE while attending to these admitted patients. There is another team in reserve which is called the rest team who is always ready to replenish the frontline (ED or Ward and ICU) depending on the need. A fourth team which is the COVID-19 team was created when there was a sharp increase in the number of COVID-19 positive trauma victims. This COVID-19 team takes care for only the COVID-19 positive patients who admitted at a nearby dedicated facility. They don a Level 3 PPE and have been responsible for the immediate as well as definitive care for the patients. All the HCWs are being rotated through all workstations weekly to maintain their physical and mental wellbeing.

**Fig. 4:**
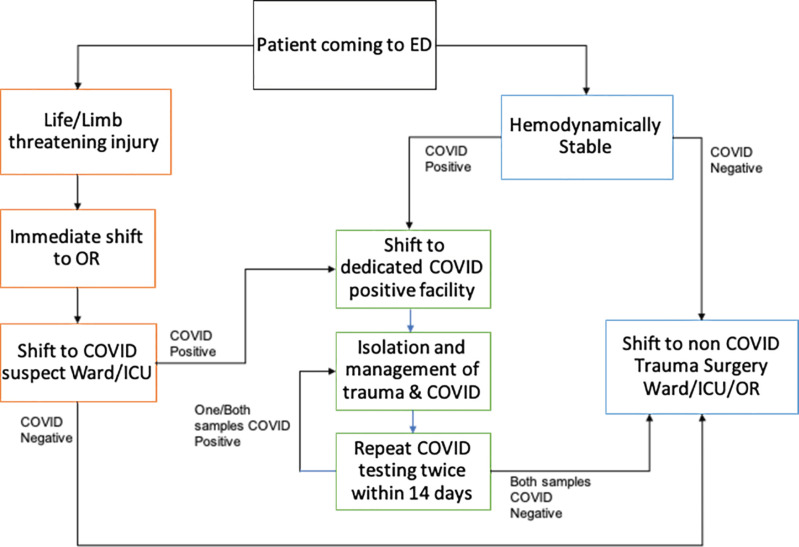
Algorithm showing the flow of trauma patients between facilities during the COVID-19 period

The work flow designing in the Trauma ED has been crucial for minimizing the risk of exposure among healthcare professionals as well as cross infections among patients. Figure 4 shows the patients’ flow in the trauma emergency department at our Level-I trauma center.

## Discussion

The patients have no choice but to visit hospitals for acute diseases like trauma/ injuries and unlike elective medical conditions despite to the potential of getting infected with the Novel Corona Virus. As the COVID-19 individuals’ infection are increasing exponentially, it is imminent to generate the reliable data of trauma volumes and injury patterns to guide health care facilities for planning the remaining course of the current and future local/ global health care emergencies. 

This study found a significant reduction in emergency trauma services’ visits, traffic injuries, and hospital admissions number. Like many countries around the world, the Indian government imposed lockdown and travel ban in anticipation of flattening the curve. These measures are very crucial because no healthcare system can sustain a massive influx of highly infectious cases with unprepared hospitals and health care infrastructure. The lockdown period gave the time for necessary preparations for gearing up the health systems for such an unprecedented situation.

Jorge *et al*., [3] reported a significant reduction in an emergency trauma visits number that leading to approximately one-fourth as many trauma admissions as the pre-pandemic period. There were no statistically significant differences in the mechanism of injuries though decreases in labour and traffic accidents were observed. 

In the present study, there was a relative increase in the patients’ triaged percentage as red (critically injured). It holds significant implications for further planning while reorganizing clinical care areas in such pandemics, resources should be adequately allotted to trauma care areas especially in the ED, clearly demarcating the areas for different triage categories. 

Christey *et al*., [4] from New Zealand reported an overall decrease in the admission of 43 %. They reported a reduction in both moderate (ISS<12) and major (ISS>12) injuries. In this study, there was a 50 % decrease in men patients. In the present analysis, there was a percentage reduction in patients with major injuries but moderate injuries were increased and there was no gender-based reduction.

In our study, there was no significant difference across different age groups unlike other studies which analysed all emergency admissions in the older age group with higher admissions [5]. It can be explained as our study was exclusively on trauma patients in a country with 65 % population below 35 years of age. 

The RTIs incidence were decreased but not as much expected during a nationwide lockdown. This might have been due to the fact that the freight traffic was still running for essential services and there was a reduction in traffic density that lead to higher speeds of vehicles and the general non-compliance of the lockdown by the society. There was also a considerable rise in interpersonal violence/assault incidence during the lockdown period which might be due to the involvement of law enforcement agencies in the COVID-19 lockdown instead of their normal work. This further stresses the need for maintaining law enforcement more stringently as during the non-lockdown period.

There was an increase in the falls’ number as compared to the pre-lockdown period. People during the lockdown were forced indoors and were involved in household activities themselves and attribute to the increase in falls from height, stairs, ladders, etc. This further substantiates need for enforcing injury prevention awareness in the society especially in high risk groups like children and senior citizens. 

The percentage of patients who were got primary care before their referral to Level-1 centre were reduced as compared to the pre-lockdown period during the lockdown. The trauma care paradigm is worsens in a country like India where primary care is already lacking. There is a need to prepare guidelines for peripheral health care facilities regarding providing emergency care and following transfer protocols especially during widespread health care emergencies and pandemics.

In post COVID-19 pandemic, few reports had suggested a high mortality in surgical patients and based on this various guidelines have come up recommending minimum surgical interventions during this pandemic [6]. These recommendations cannot possibly be applicable to trauma care where emergent and urgent surgeries are essential for life and limb salvage. The outcome parameters were unchanged, although we had a higher number of patients (25%) going directly to OT from ED as compared to 19% during the pre-lockdown period. We are suggesting that dedicated trauma services are crucial to maintain quality of trauma care with modified in-hospital care systems in widespread health emergency settings like pandemics. 

Our data shows that 12% of general trauma patients were found COVID-19 positive after initial assessment and management in the ED. This makes ED staff at higher risk than other HCWs and justifies the need to have the highest level of protection for ED staff. 

Some of the other significant systems issues were faced during the lockdown and post COVID-19 period includes workforce sensitization for COVID-19 protection, protocol adherence, blood product shortage, shortage of workforce (due to exposure or getting infected), OT’s readiness for emergent surgeries in patients with unknown COVID-19 status, slow turnover times for COVID-19 tests from ED etc. These issues were sorted out with multi-stakeholder involvement, repeated information, education and communication (IEC) activities, and continuous quality of care monitoring through frequent “closure of loop” through active administrative oversight.

In summary, it is obvious that the injuries’ incidence would not reduce significantly even during health care emergencies like pandemics and they might show a decline during periods of general lockdown but will catch up fast when such lockdowns are relaxed. This must be kept in mind while developing contingency plans and reallocating resources during such health emergencies, epidemics or pandemics. This study highlighted that the preparedness should not focus solely on the response to treat infectious disease but also on ensuring access and provision of reasonable quality of care for non-infectious illnesses especially acute conditions like trauma.

Acute emergencies like trauma continue to burden to health care system as was seen in even in a pandemic situation like COVID-19, therefore, all attempts should be made not to disturb the existing trauma care facilities for treating COVID-19/COVID-19 like illnesses. The authors feels that isolation facilities for infectious pandemic like COVID-19 should be decentralized and managed by individual specialties and the COVID-19 individuals can be managed properly but the primary disease for which the patient has been admitted and can be catered to by specialists. Trauma ED’s should have contingency plans including resource allocation (PPE kits/rapid diagnostic modalities etc.) and standard operating procedures (SOPs) highlighting that all cases coming to the hospital be treated as suspected positive cases of the COVID-19/infectious pandemic.

Trauma and injuries causes significant derangement of physiology and consequent immunological derangements and since, COVID-19 is an infectious disease that causing variable immune response, further studies should also be directed towards studying the combined immunological responses in trauma individuals admitted with concomitant COVID-19 / COVID-19 like infections.

## Limitation 

This study did not examine the care processes for trauma patients and its impact on key performance indicators affecting the overall quality during the pandemic. It is perceived that the treatment timelines would show some prolongation due to human as well as system issues such as arrival time, initial assessment and management time, X-Ray/ computed tomography (CT) time, OT’s time and etc., which might affect trauma outcomes. There is also a need for analysing data from other trauma care facilities levels to see the effect of the pandemic on the utilization of trauma facilities across the full spectrum of the trauma care system.

## Ethics Approval:

Ethics approval was obtained from the institute ethics committee of AIIMS for the project (Ref No- IEC-294/03.05.2019, RP-52/2019).

## Financial support:

The present study was conducted using trauma registry data which is a part of the project financially supported by the Indian Council of Medical Research (ICMR), New Delhi (Ref No-5/4-5/3/7/Trauma/2019-NCD-I).

## Acknowledgment:

Not applicable.

## Conflict of Interests:

None declared.
